# Brain Cancer Chemotherapy through a Delivery System across the Blood-Brain Barrier into the Brain Based on Receptor-Mediated Transcytosis Using Monoclonal Antibody Conjugates

**DOI:** 10.3390/biomedicines10071597

**Published:** 2022-07-05

**Authors:** Toshihiko Tashima

**Affiliations:** Tashima Laboratories of Arts and Sciences, 1239-5 Toriyama-cho, Kohoku-ku, Yokohama 222-0035, Japan; tashima_lab@yahoo.co.jp

**Keywords:** brain cancer chemotherapy, antibody-drug conjugates, drug delivery system, drug delivery into the brain across the BBB, receptor-mediated transcytosis, transferrin receptor-mediated endocytosis, anti-TfR ADCs with cancer drugs, pH-sensitive cleavable linkers, anti-TfR and anti-EGFR bispecific ADCs with payloads, state-of-the-art biomedicines

## Abstract

Advances in pharmacotherapy have brought extraordinary benefits to humanity. However, unmet medical needs in patients remain, particularly in the treatment of central nervous system (CNS) diseases and cancers. CNS drug delivery into the brain across the endothelium is difficult due to the blood-brain barrier (BBB), which is composed mainly of tight junctions and efflux transporters, such as multiple drug resistance 1 (MDR1) (P-glycoprotein). On the other hand, the development of anti-cancer drugs is a challenging task due to their frequent off-target side effects and the complicated mechanisms of cancer pathogenesis and progression. Brain cancer treatment options are surgery, radiation therapy, and chemotherapy. It is difficult to remove all tumor cells, even by surgical removal after a craniotomy. Accordingly, innovative brain cancer drugs are needed. Currently, antibody (Ab) drugs that show high therapeutic effects are often used clinically. Furthermore, antibody-drug conjugates (ADCs), such as trastuzumab deruxtecan, an anti-HER2 (human epidermal receptor 2) ADC with low-molecular cancer drugs through the suitable linker, have been developed. In the case of trastuzumab deruxtecan, it is internalized into cancer cells across the membrane via receptor-mediated endocytosis. Moreover, it is reported that drug delivery into the brain across the BBB was carried out via receptor-mediated transcytosis (RMT), using anti-receptor Abs as a vector against the transferrin receptor (TfR) or insulin receptor (InsR). Thus, anti-TfR ADCs with cancer drugs are promising brain cancer agents due to their precise distribution and low side effects. In this review, I introduce the implementations and potential of brain cancer drug delivery into the brain across the BBB, based on RMT using ADCs.

## 1. Introduction

**Figure 1:**
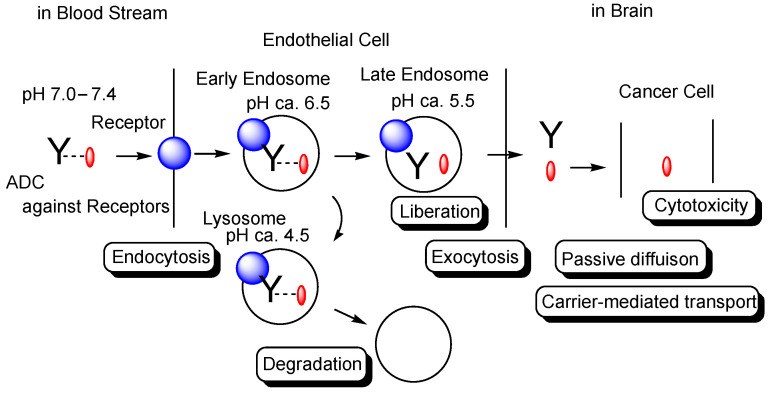
The pathway of intravenously administered antibody-drug conjugates (ADCs) against receptors such as the transferrin receptor (TfR), toward exhibiting brain cancer cell cytotoxicity through receptor-medicated transcytosis (RMT) in the secretory pathway. The mAb-TfR complex was liberated under weakly acidic conditions in the endosomes. Furthermore, linked drugs acting as a payload were also liberated via the cleavage of pH-sensitive cleavable linkers under weakly acidic conditions in the endosomes. Drugs released into the brain parenchyma can be transported into cancer cells and can show anti-cancer activity. Y represents a monoclonal antibody (mAb). The blue sphere indicates a receptor that mediates transcytosis in the capillary endothelial cells at the blood-brain barrier. The red ovals represent a drug that is tethered with a mAb through a suitable linker. The dotted line indicates a linker contained in an ADC. The solid line represents the membrane.

It is true that medical treatment has brought a significant benefit to human health, but unmet medical needs still remain, particularly in the treatment of central nervous system (CNS) diseases [1] and cancers. In particular, brain cancer drug development is, synergistically, an extraordinarily challenging task with respect to pharmacokinetics and pharmacodynamics. Drug delivery into the brain presents a serious impenetrable problem in CNS drug development, due to repulsion by the blood-brain barrier (BBB) [2,3]. Most drugs cannot be transported from the systemic circulation to the brain across the BBB. The BBB is substantially composed of (i) a physical barrier based on hydrophobic lipid bilayer membrane, (ii) a physical barrier based on tight junctions between the capillary endothelial cells, (iii) a biological barrier based on efflux transporters such as multiple drug resistance 1 (MDR1) (P-glycoprotein), and (iv) a physical barrier based on a lining supported by pericytes and astrocytes. Accordingly, intravenously administered CNS drugs must cross the BBB to complete their activity in the target sites. Receptor-mediated transcytosis (RMT) represents one of the solutions to this impenetrability problem [2]. Some receptors, such as the transferrin receptor (TfR) or insulin receptor (InsR), transport their corresponding ligands across the endothelium or the epithelium via RMT. So far, I have described several drug delivery methods across the cell membrane [2,4,5,6,7,8,9] using RMT or carrier-mediated transport, based on rational drug design, particularly across the BBB [2,9]. From such an investigative process, it has been revealed that pharmacokinetically effective drug delivery can be accomplished by rigorous design, regulated through physically and biologically systematic structures such as the BBB or RMT machinery system, based on the theory of structuralism proselytized by Lévi-Strauss. Compounds are divided into three categories, that is, low-molecular compounds (molecular weight (MW) < approximately 500), high-molecular compounds (MW > approximately 3000), and middle-molecular compounds (MW from approximately 500 to approximately 3000). In general, while hydrophobic low-molecular compounds penetrate the cell membrane through passive diffusion, hydrophilic low-molecular compounds penetrate it via carrier-mediated transport, using solute carrier (SLC) transporters. Water-soluble low-molecular nutrition, such as glucose and amino acids, are transported into the brain across the BBB by SLC transporters expressed at the BBB. High- or middle-molecular compounds penetrate the cell membrane through receptor-mediated endocytosis, lipid-raft mediated endocytosis, or macropinocytosis. High- or middle-molecular compounds cannot physically pass through the pores of SLC transporters due to their molecular size, although they can pass through transient disruption of tight junctions in the BBB. Bystander low-molecular compounds in the bloodstream are internalized into endosomes after spontaneous and receptor-mediated endocytosis, although they cannot induce endocytosis. Low-molecular compounds are subject to enzymatic metabolism and to excretion by kidneys into urine and by liver into bile. A drug delivery strategy can be naturally established, depending on which class of compounds in terms of size are used, in addition to their hydrophobicity and hydrophilicity. In this perspective review, I will introduce the topic of the delivery of brain cancer drugs across the BBB into the brain based on RMT, using antibody-drug conjugates (ADCs) in terms of state-of-the-art biomedicines (Figure 1). I apologize in advance for the fact that this article was written for the Special Issue entitled “State-of-the-Art Cancer Biology, Biodiagnostics, and Therapeutics in Japan”. Thus, Japanese technologies have been picked up in a relatively limited way, reflecting global trends. To complement global trends further, the readers may refer to other review articles that are cited in this article or those that are suggested by search tools. Nonetheless, current trends in drug delivery can be acquired from this article.

## 2. Discussion

### 2.1. Brain Cancers and Their Chemotherapy

Cancer is a leading cause of death. Brain cancers are intracranial neoplasms that are either primary tumors or metastasizing tumors, occurring in approximately one hundred cases per one million people. They are broadly categorized into glioma, metastatic brain tumors, medulloblastoma, malignant lymphoma, germ cell tumors, meningioma, hypophyseal adenoma, and neurilemmoma. Treatments for brain cancers impose burdens on patients due to headaches, the consequences of surgical removal after craniotomy, and side effects from chemotherapy or radiation therapy. Thus, innovative brain cancer therapy is urgently needed to improve patients’ quality of life.

Glioma [10], derived from glial cells, currently accounts for approximately one-third of brain cancers in Japan. The surgical removal of brain tumors, in some cases by labeling tumor cells with 5-aminolevulinic acid, is performed as a standard of care. Nonetheless, it is difficult to remove all tumor cells. Consequently, brain cancer chemotherapy, particularly for glioma, is generally conducted after tumor removal.

**Figure 2:**
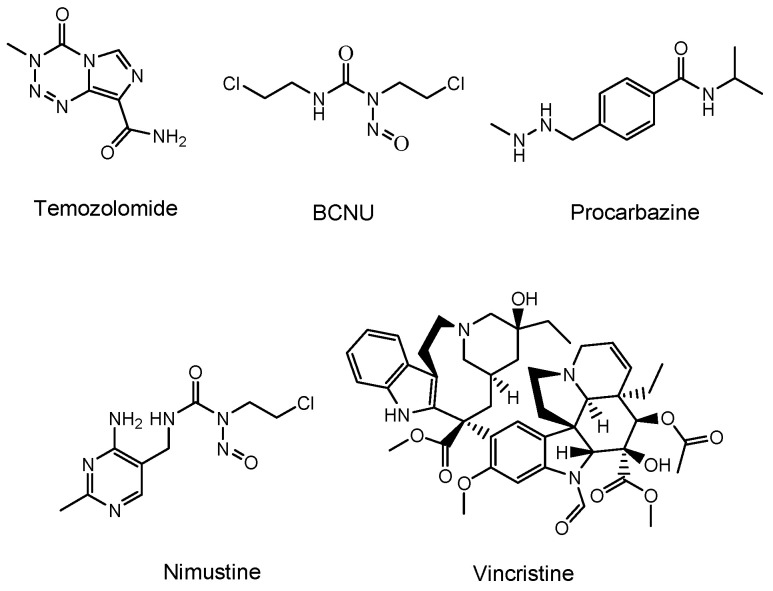
Structures of low-molecular drugs that are used clinically for brain cancers.

Temozolomide (an alkylating agent) [11], bevacizumab (a monoclonal antibody (mAb) against vascular endothelial growth factor (VEGF) that is relevant in angiogenesis and is released from cancer cells) [12], and a BCNU wafer (an alkylating slow-release agent) [13] are often clinically prescribed for glioma (Figure 2). Temozolomide is so hydrophobic that it penetrates the cell membrane via passive diffusion. However, MDR1 at the BBB captures hydrophobic low-molecular compounds that are just passing through the capillary endothelial cell membrane and excretes them to the systemic circulation. Thus, most of the temozolomide might be excreted by MDR1 at the BBB, without being distributed to the brain. Moreover, mAbs are so large and so hydrophilic that they cannot penetrates the cell membrane via passive diffusion. Thus, mAbs such as bevacizumab cannot penetrate the capillary endothelial cell membrane at the BBB, without being distributed to the brain. Furthermore, a BCNU wafer composed of polifeprosan 20, a degradable polymer, is left in the brain after tumor removal. However, the pharmacokinetics of polifeprosan 20 in the human brain remains unclarified, although, in the rat brain and rabbit brain, this was verified. Therefore, innovative drug delivery approaches should be developed, in order to carry out effective and harmless chemotherapy treatment.

### 2.2. Possibility and Implement of ADCs

Historically, biomedicines using mAbs have followed the genealogy from mAb drugs to ADCs, and further to bispecific mAbs. To deliver drugs to the brain across the BBB, the technologies of bispecific mAbs or bispecific ADCs through RMT are needed. In the future, trispecific mAbs will become a practical possibility. I will describe mAb biomedicines below, according to such a genealogy.

#### 2.2.1. mAb Drugs

**Figure 3:**
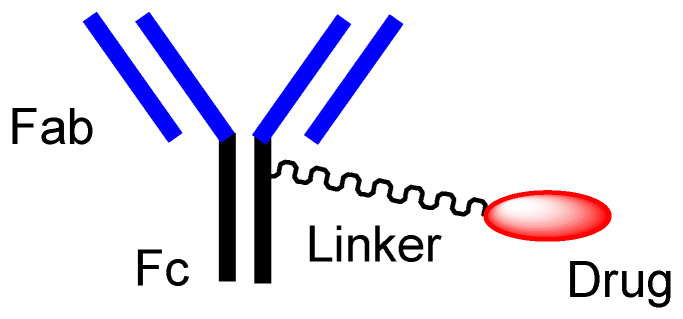
The typical structure of antibody-drug conjugate (ADC).

First of all, mAbs exhibit highly selective binding to the epitopes in their target antigen molecules. Ab drugs, as molecular targeted drugs, are representative biomedicines [14]. An Ab protein, such as immunoglobulin G (IgG), is structurally constituted of two heavy chains and two light chains and is enzymatically cleaved by papain into a fragment antigen-binding (Fab) region and a fragment crystallizable (Fc) region (Figure 3). Japanese mAb technologies are excellent, and outstanding mAbs have been developed. Representative Ab production technologies include Ab manufacturing technology with high Ab-dependent cellular cytotoxicity (POTELLIGENT^®^) [15] by Kyowa Kirin (Tokyo, Japan), recycling Ab manufacturing technology (SMART-Ig^®^) [16] by Chugai (Tokyo, Japan), and bispecific Ab manufacturing technology (ART-Ig^®^) by Chugai. Tocilizumab (Actemra^®^) [17], developed as an anti-IL-6 mAb by Chugai, was approved for rheumatoid arthritis treatment in 2010 by the US Food and Drug Administration (FDA). Nivolumab (Opdivo^®^) [18], developed as an anti-PD-1 (programmed cell death 1) mAb by Ono (Osaka, Japan), was approved for metastatic lung squamous cell carcinoma treatment in 2014 by the FDA. Mogamulizumab (Poteligeo^®^) [19], developed as anti-CCR4 (CC chemokine receptor 4) mAb using POTELLIGENT^®^ by Kyowa Kirin, was approved for the treatment of relapsed or refractory mycosis fungoides and Sézary disease in 2018 by the FDA. Burosumab (Crysvita^®^) [20], developed as an anti-FGF23 (fibroblast growth factor 23) mAb by Kyowa Kirin, was approved for treating X-linked hypophosphatemic rickets in 2018 by the FDA.

#### 2.2.2. Orthodox ADCs

**Figure 4:**
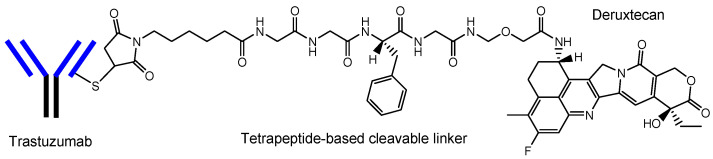
The structure of trastuzumab deruxtecan (Enhertu^®^), with a drug-to-antibody ratio (DAR) of 7.7.

ADCs comprise another area of development. The high selectivity of mAb enables specific distribution in terms of drug delivery. Thus, at present, ADCs, composed of an antibody as a vector and the drug as a payload via suitable linkers (Figure 3), have attracted a great deal of attention [6,21]. In the case of cancer therapies, antibody-cancer drug conjugates are thought to reduce off-target side effects, such as normal hematopoietic cell damage or hair loss, due to the specific interaction between epitopes as receptors that are specifically expressed on target cancer cells and mAbs as ligands, and due to the non-interaction between off-target molecules and linked cancer drugs, based on the bulkiness and large size of mAb. Such bulkiness might offer an escape from enzymatic drug degradation. If the Fc region of mAb is not occupied with payloads or linkers, the half-life of ADCs might elongate, based on salvation by the neonatal Fc receptor (FcRn) at the endothelium [8,22]. Actually, ADCs acting against the surface epitopes on cancer cells have been clinically utilized for solid cancers such as lung cancer, colon cancer, or breast cancer, and for blood cancers such as acute myelogenous leukemia. Trastuzumab deruxtecan (Enhertu^®^) (Figure 4) [21], an anti-HER2 (human epidermal receptor 2) ADC developed by Daiichi Sankyo (Tokyo, Japan), was approved for HER2-positive breast cancer in 2019 by the FDA. Deruxtecan is a DNA topoisomerase I inhibitor. Trastuzumab deruxtecan was internalized into cancer cells via receptor-mediated endocytosis, using HER2 [23]. Furthermore, Daiichi Sankyo has developed (i) Dato-DXd as an anti-ROP2 ADC [24], (ii) HER3-DXd as an anti-HER3 ADC [25], (iii) DS-7300 as an anti-B7-H3 ADC [26], (iv) DS-6000 as an anti-CDH6 ADC [27], and (v) DS-3939 as an anti-TA-MUC1 ADC [27], for solid cancers, respectively. Accordingly, ADCs might be ideal for use in brain cancer therapy, with respect to their effective distribution to the brain and low off-target side effects [6]. However, surprisingly, ADCs for brain cancer have not been developed, probably due to poor distribution because of the BBB.

#### 2.2.3. Anti-Receptor ADCs That Cross the Endothelium via RMT

**Figure 5:**
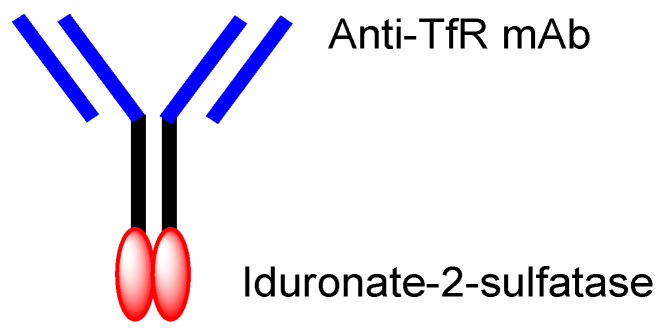
The structure of idursulfase beta, composed of anti-TfR (transferrin receptor) mAb (monoclonal antibody) and iduronate-2-sulfatase.

The enhanced permeation and retention (EPR) effect, discovered by Dr. Yasuhiro Matsumura and Dr. Hiroshi Maeda in 1986, spontaneously accumulates nanoparticles in cancer tissues, due to leaky blood vessels and hypervascularization [6,28]. However, this cannot be expected in brain cancers due to the tight junctions at the BBB, although part of the membranes or tight junctions at the BBB might be disrupted as the result of pathological alteration. It is well known that RMT at the BBB, using TfR or InsR, is used for CNS drug delivery into the brain. Anti-TfR mAbs are endocytosed at the apical membrane of the capillary endothelial cells after ligand-receptor binding, are then liberated from TfRs through acidification in the endosomes, and are, finally, released into the brain via exocytosis, based on the fusion between the endosomes and the basolateral membrane. Moreover, mAbs acting as ligands showed higher selectivity than the cell-penetrating peptides (CPPs) such as TAT (YGRKKRRQRRR) and penetratin (RQIKIWFQNRRMKWKK). CPPs are positively charged short peptides, with 5–30 amino acids, that cross the cell membrane through receptor-mediated endocytosis or by direct translocation [5]. Although TAT, as a ligand, induced receptor-mediated endocytosis using the negatively charged heparan sulfate proteoglycans (HSPGs) on the cell surface, HSPGs in the form of receptors are ubiquitously expressed on many types of cells. Interestingly, J-Brain Cargo^®^ (Figure 5) [2,29], established by JCR Pharmaceuticals (Ashiya, Japan), is a system to deliver drugs into the brain across the BBB via RMT, using ADCs composed of anti-TfR mAb and drugs. This system is used by the drug idursulfase beta (Figure 5), approved in Japan on 23 March 2021 for the treatment of Hunter syndrome [30]. Takeda (Tokyo and Osaka, Japan) is going to sell idursulfase beta worldwide, together with JCR Pharmaceuticals. The concept idea of idursulfase beta, based on J-Brain Cargo^®^, can be technologically adapted to cancer therapy by replacing the payloads. Once ADCs are transported into the brain parenchyma via RMT, they or their liberated payloads can stay there without being excreted out of the brain by the BBB. This feature might help to reduce off-target side effects outside the brain.

#### 2.2.4. Anti-Receptor ADCs including Bispecific ADCs

**Figure 6:**
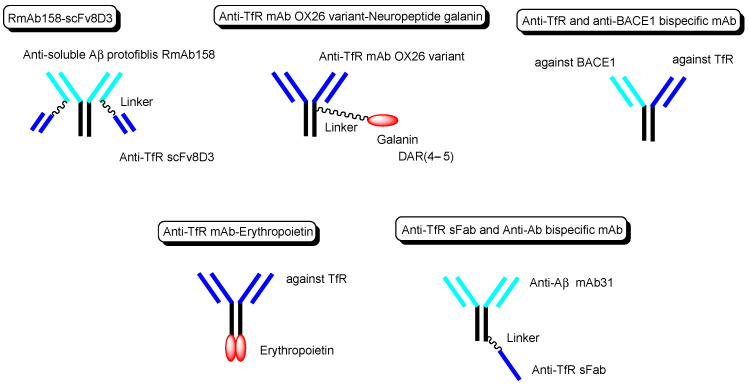
The structures of anti-TfR mAbs, conjugated to active cargos.

Other technical cases of drug delivery across the BBB via RMT using anti-TfR mAbs as a vector are shown here, including anti-receptor and anti-target molecule bispecific technologies. (i) Bispecific RmAb158-scFv8D3 (Figure 6) is a conjugate of scFv8D3 with two single-chain (sc) variable fragments of anti-TfR Ab 8D3 and RmAb158 against soluble amyloid β (Aβ) protofibrils, through the linker at each *C*-terminus of scFv8D3, and two light chains of RmAb158. Actually, [^125^I]-RmAb158-scFv8D3 was transported 80-fold higher in the brain than [^125^I]-RmAb158 in an in vivo assay with mice euthanized 2 h after intravenous injection [31]. (ii) The conjugate of the anti-TfR mAb OX26 variant and neuropeptide galanin (YPSKPDNPGEDAPAEDMARYYSALRHYINLITRQRY), with a drug-to-antibody ratio (DAR) of 4–5 at cysteine residues on the Fc region of the OX26 variant through a maleimide group (Figure 6), inhibited thermal hyperalgesia in an in vivo assay intravenously administered in a rat model, compared to the conjugate of galanin and control mAb NiP228 [32]. (iii) Intravenously administered anti-TfR and anti-BACE1 (β-amyloid cleaving enzyme-1) bispecific mAb (Figure 6) reduced brain Aβ levels in an in vivo assay using mice. [33]. (iv) Anti-TfR mAb, when fused to erythropoietin (Figure 6), significantly lowered cortical and hippocampal Aβ peptide levels, decreased hippocampal synaptic loss, decreased cortical microglial activation, and improved spatial memory in an APP/PS1 mouse model of Alzheimer’s disease (AD) [34]. (v) The monovalent conjugate of one scFab fragment of an anti-TfR mAb and anti-Aβ mAb31, linked at one *C*-terminal end of the heavy chain of anti-Aβ mAb (Figure 6), was transported into the brain across the BBB via RMT and exhibited 55-fold higher Aβ plaque binding than the parent mAb31 after intravenous administration, in an in vivo assay using PS2APP transgenic mice [35]. Interestingly, the bivalent conjugate of two scFab fragments of an anti-TfR mAb and anti-Aβ mAb31, linked at one *C*-terminal end of the heavy chain of anti-Aβ mAb31 with one scFab fragment for each, bound simultaneously to two TfRs and subsequently degraded as the generated complex in lysosomes after receptor-mediated endocytosis. Incidentally, bispecific RmAb158-scFv8D3 bound one TfR due to structural restriction and was endocytosed, although it had two scFv8D3 [31].

Nonetheless, the orthodox type of anti-TfR ADCs, with linked low-molecular brain cancer drugs (Figure 3), is not likely to be developed. Therefore, well-designed brain cancer drug delivery into the brain via RMT, using anti-receptor mAbs as a vector, has great possibilities.

### 2.3. Activity Expression of ADCs

Activity expression by the payloads of ADCs assumes two scenarios, such as (i) the liberation of payloads after linker cleavage, or (ii) no structural alteration of ADCs, including bispecific mAbs or ADC with relatively bare payloads, such as idursulfase beta. If low-molecular cancer drugs were conjugated as a payload, they would be cut off from ADCs due to the bulkiness of mAb, shown in the curious case of AMG-595, below. Several linker cleavage systems have been developed [6]: (a) pH-sensitive cleavable linkers, such as hydrazone, which can be cleaved in an acidic endosome, (b) reductively cleavable linkers, such as the disulfide bond, (c) enzymatically cleavable linkers, such as valine-citrulline dipeptide, (d) self-immolative linkers, such as the para-aminobenzyloxycarbonyl group, and (e) other mechanistically cleavable linkers, such as a photocleavable component.

The extracellular physiological pH is between 7.0 and 7.4. The pH in endosomes gradually reduces from the early endosome (pH of approximately 6.5) to the late endosome (pH of approximately 5.5) and, finally, lysosome (pH of approximately 4.5) by vacuolar H^+^-ATPase proton pumps. Thus, ideally, the linker of the anti-TfR mAb-cancer drug conjugate should be cleaved in endosomal acidification. In fact, pH-sensitive cleavable linkers were developed. At the same time, anti-TfR mAbs should be liberated from TfRs under weakly acidic conditions, without being degraded in lysosomes in the degradation pathway. When anti-TfR mAbs bind TfR too tightly, the resulting unliberated complexes are degraded into pieces in lysosomes by the lysosomal enzymes. Actually, the affinity of anti-TfR mAbs to TfR can be tuned so as to be separated as endosomal acidification. Eventually, free cancer drugs are released into the brain after exocytosis in the secretory pathway (Figure 1) [2]. The released drugs move within the brain parenchyma, due to restricted distribution by the BBB and, subsequently, elicit their activity in target sites on the surface of cancer cells or in cancer cells after internalization via passive diffusion or carrier-mediated transportation. Intriguingly, the FcRn-IgG complex is formed in the early endosomes because FcRn binds the Fc region of IgG under weakly acidic conditions (pH < 6.5) [8]. The FcRn-IgG complex is exocytosed to the systemic circulation in the secretory pathway and is liberated under extracellular physiological pH. This system by FcRn at the endothelium results in the long half-life of ADCs, due to their salvation from lysosomal degradation. Therefore, drug designs should be carried out in the context of the involved physically and biologically systematic structures.

On the other hand, reductively cleavable linkers, enzymatically cleavable linkers, and other mechanistically cleavable linkers, such as a photocleavable component, should be cleaved in the brain parenchyma. Orthodox ADCs that had already crossed the BBB via RMT would probably not enter the brain cancer cells via RMT again; therefore, they might release their payloads in the brain parenchyma.

### 2.4. Promising Implement of ADCs for the Treatment of Brain Cancers

Regional BBB disruption is often invoked by brain cancers but it is a biologically transient and inconstant environment, depending on the pathology, physical condition, and individual variability. VEGFs secreted from brain cancer cells induced angiogenesis, lost the astrocyte endfeet, and destroyed the tight junctions to form fenestration (up to 15 nm in diameter between common endothelial cells, potentially including the BBB [36,37]). Even such a BBB scenario, called the brain–tumor barrier, restricted material permeation to some extent, which permeation was conducted through the paracellular fenestration pathway and transcellular pinocytosis [38,39]. It seems likely that brain cancers substantially occupy the endothelial cells while alive, some of which are established by VEGF-induced angiogenesis, to deliver themselves nutrients. The EPR effect, effective for molecules that are 10–100 nm in diameter, was not expected in the open tight junction pores at the BBB with the exception of an injured BBB, because the IgG protein used for ADCs is approximately 14.2 nm in diameter [8]. Open tight junction pores in the olfactory epithelium were expanded intentionally up to approximately 15 nm by permeation enhancers [7]. In addition, olfactory sensory neurons are subject to injury or cell death, due to their exposure to exogenous materials, which induced an injured, leaky olfactory epithelium [7]. A small amount of RmAb158 without the anti-TfR vector (Figure 6) was distributed into the brain through the damaged BBB in AD in an in vivo assay using a mouse model [2]. Aducanumab penetrated dose-dependently into the brain through the damaged BBB, caused by an aducanumab-induced microhemorrhage [9]. Nonetheless, even though drugs such as mAb drugs were intravenously administered, regional BBB disruption, such as tight junction destruction, and the endothelial cell death-induced injury to the BBB by brain cancers would not be always ready to accept the drugs in favorable conditions, due to a transient and inconstant environment. Thus, the RMT strategy for transendothelium at the BBB, using anti-receptor mADCs, is a preferable tactic.

Moreover, cancer-derived exosomes to the capillary endothelial cells at the BBB, acting as recipient cells, induced the disruption of the BBB by destroying tight junctions before metastasis, which reflected the “seed and soil” theory by Dr. Stephen Paget [2]. However, glioma is not metastatic cancer but is instead a primary cancer derived from glial cells. Probably for that reason, it was revealed that all glioblastomas, which are highly malignant gliomas, do not disrupt the BBB [40]. Furthermore, not all brain cancer cells are removed by surgical removal after craniotomy. Prognostic chemotherapy should be continued. Thus, a drug delivery system into the brain across the undisrupted BBB for the treatment of glioma is needed, because mAbs and hydrophobic low-molecular compounds cannot usually cross the undisrupted normal BBB and the regenerated BBB.

**Figure 7:**
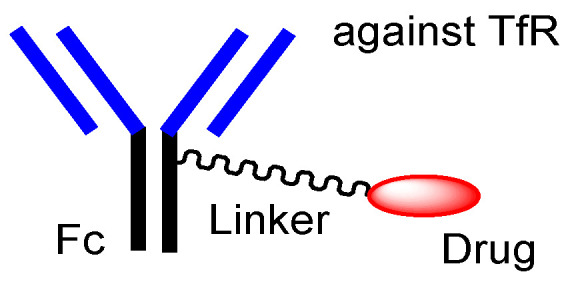
The structures of anti-TfR (transferrin receptor) antibody-drug conjugate (ADC), containing cancer drugs via pH-sensitive cleavable linkers.

**Figure 8:**
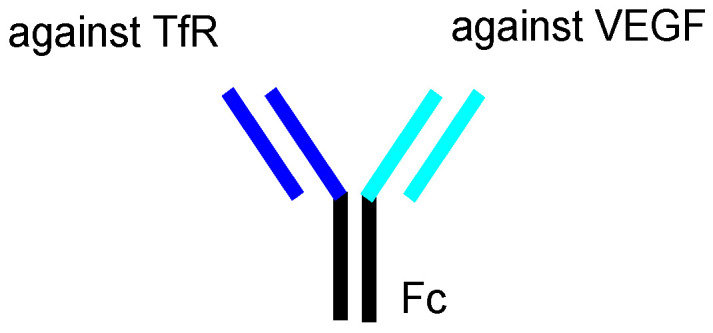
One of the structures of bispecific IgG against transferrin receptor (TfR) and vascular endothelial growth factor (VEGF).

To the best of my knowledge, there are few or are not any ADCs for brain cancer, even at the basic research level. Intravenously administered anti-TfR ADCs containing low-molecular cancer drugs with weak pH-sensitive, cleavable linkers can be promising brain cancer drugs (Figure 7). If the anti-TfR mAb-TfR binding has a high affinity, this complex is not separated as endosomal acidification and is eventually degraded together in the lysosomes on the degradation pathway, without being exocytosed [2]. Thus, anti-TfR mAb-TfR binding must have a moderate affinity, to be subjected to the secretory pathway. Alternatively, anti-TfR and anti-VEGF bispecific mAbs, such as the hybrid of an anti-TfR mAb as a vector, and bevacizumab as a payload, are possible candidates (Figure 8). Nonetheless, anti-TfR and anti-VEGF bispecific mAbs would reduce the VEGF levels in the brain parenchyma and would eventually inhibit the proliferation of cancer cells. According to the circumstances, the co-administration of other anti-cancer drugs might be needed to kill brain cancer cells effectively.

**Figure 9:**
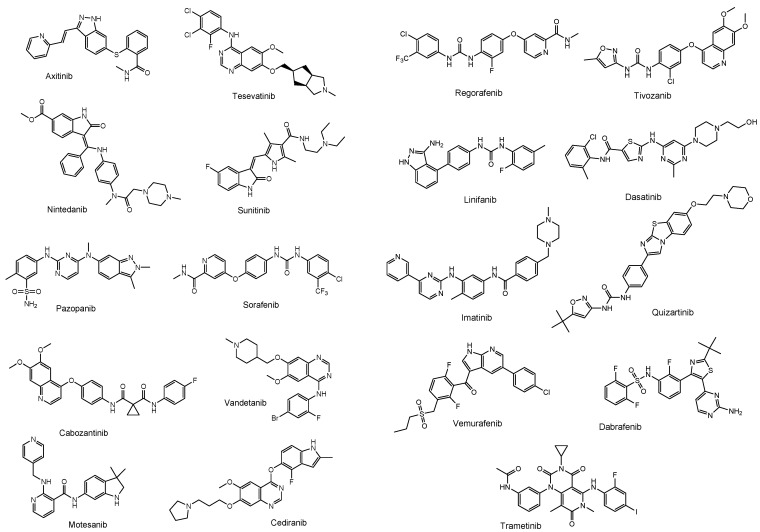
The structures of low-molecular drugs that are potential vascular endothelial growth factor (VEGF) inhibitors.

**Figure 10:**
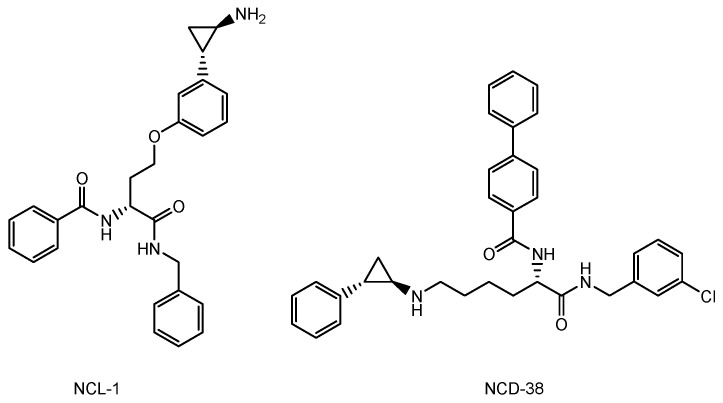
The structure of lysine-specific demethylase-1 (KDM1A) inhibitors such as NCL-1 and NCD-38.

The drugs currently used for brain cancers are temozolomide [11], bevacizumab as the mAb VEGF inhibitor [12], BCNU (bis-chloroethylnitrosourea) (carmustine), contained in a BCNU wafer [13], procarbazine [41], nimustine (ACNU) [42], developed by Daiichi Sankyo, and vincristine [43]. Because procarbazine in the form of a prodrug is oxidized in the liver to change into its active form, it is not suitable for intravenously administered ADCs in this case. Low-molecular drugs that may be potential VEGF inhibitors are: axitinib, tesevatinib, nintedanib, sunitinib, pazopanib, sorafenib, cabozantinib, vandetanib, motesanib, cediranib, regorafenib, tivozanib, linifanib, dasatinib, imatinib, quizartinib, vemurafenib, dabrafenib, and trametinib [44] (Figure 9). Therefore, payloads should be chosen among drugs such as temozolomide, BCNU, nimustine, vincristine, the VEGF inhibitors shown above, and other known cancer drugs, such as lysine-specific demethylase-1 (KDM1A) inhibitors (Figure 10), which induced the differentiation and apoptosis of glioma stem cells, in a treatment developed by Dr. Takayoshi Suzuki at Osaka University (Suita, Japan) [45]. The introduction of functional groups, such as the hydroxy group, to connect the suitable linker tethered with mAbs can be conducted into the inactive region of low-molecular drugs, which are different from their pharmacophore.

**Figure 11:**
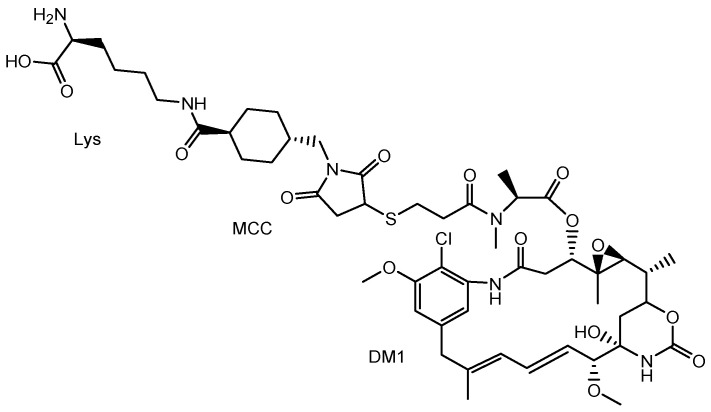
The structure of lysine-MCC-DM1.

**Figure 12:**
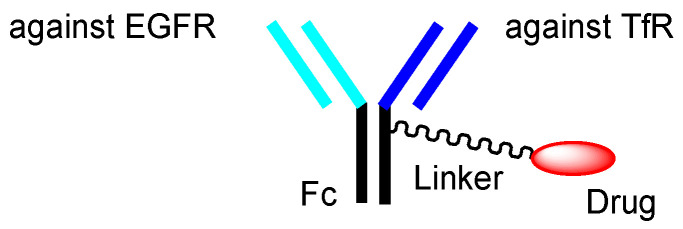
The structure of anti-TfR (transferrin receptor) and anti-EGFR (epidermal growth factor receptor) bispecific ADC with low-molecular payloads.

It is known that anti-EGFR (epidermal growth factor receptor) ADC, with monomethyl auristatin F (MMAF) as a tubulin polymerization inhibitor, via a non-cleavable maleimidocaproyl (MC) linker, ABT-414 (depatuxizumab mafodotin) [46], and anti-EGFR ADC with DM1 (mertansine) as a tubulin polymerization inhibitor, via a non-cleavable maleimidomethylcyclohexane-1-carboxyl (MCC) linker, AMG-595 [47], exhibited high antitumor activity in preclinical glioblastoma tumor models [48]. Highly malignant, grade-IV glioma is often known as glioblastoma. The tubulin polymerization inhibitors of ABT-414 and AMG-595 are thought to show activity in cancer cells after receptor-mediated endocytosis. Cancer cells, including preclinical glioblastoma tumor models, express EGFR. In particular, the EGFR variant III (EGFRvIII) is expressed in cancer cells. AMG 595, bearing anti-EGFRvIII Ab, was internalized into cancer cells via receptor-mediated endocytosis, using EGFRvIII [47]. However, it is uncertain whether ABT-414 and AMG-595 cross the BBB, due to the lack of a vector unit after intravenous administration. In general, mAbs cannot cross the BBB due to their large size and hydrophilicity, although aducanumab, anti-Aβ mAb, which was launched in 2021 by Eisai (Tokyo, Japan), is exceptionally well-known for crossing the BBB by disturbing it [9,49]. Intriguingly, AMG-595 was enzymatically degraded into Lys-MCC-DM1 (Figure 11) as a catabolite in lysosomes [47]. The lysosomal transporter SLC46A3 transported Lys-MCC-DM1 to the cytoplasm [50]. Consequently, cytosol Lys-MCC-DM1 exhibited tubulin polymerization inhibitory activity. Lys-MCC-DM1 alone was not internalized into cells. Accordingly, bystander cytotoxicity by Lys-MCC-DM1 as a catabolite from AMG-595 cannot occur in normal cells in the vicinity. Thus, anti-TfR and anti-EGFR bispecific ADCs with low-molecular payloads through the same linkers [51] used in ABT-414 or AMG-595 (Figure 12) are also promising candidates with the fewest side effects, due to synergistic high selectivity. As an anti-EGFR mAbs, depatuxizumab is used in ABT-414 and anti-EGFRvIII Ab is used in AMG 595. Dr. Yukinari Kato at Tohoku University (Sendai, Japan) has developed several anti-human EGFR mAbs, such as EMab-51 [52] and Emab-134 [53]. Cetuximab, panitumumab, nimotuzumab, and necitumumab are already on the market as anti-EGFR mAbs. Moreover, a number of anti-EGFR mAbs are under pre-clinical studies and clinical trials [54]. Optimized anti-TfR and anti-EGFR bispecific ADCs may be obtained by choosing the appropriate anti-EGFR mAb among these options.

It is technically difficult to produce multispecific mAbs. However, recently, Dr. Hiroaki Suga at the University of Tokyo (Tokyo, Japan) and MiraBiologics (Tokyo, Japan) have developed LassoGraft Technology^®^, which is a method to introduce the active part of cyclic peptides onto the surface of other arbitrary proteins [55]. Such active cyclic peptides can be discovered via screening, through the random non-standard peptides integrated discovery (RaPID) system [56], which is based on reprogramming of the genetic code using an mRNA display developed by Dr. Suga and PeptiDream (Kawasaki, Japan). As a result, multispecific mAbs can be obtained rapidly and easily via the RaPID system and the subsequent use of LassoGraft Technology^®^. In particular, Mirabody^®^ is composed of only the Fc region, with eight grafting sites. Addbody^®^ is an Ab with more than 30 grafting sites. Although bispecific mAbs, such as the anti-blood-coagulation factor IX and anti-blood-coagulation factor X bispecific mAb emicizumab (Hemlibra^®^) [57] developed and launched for hemophilia A in 2018 in Japan by Chugai, have been clinically approved, multispecific mAbs (more than trispecific) have not yet been approved. Multispecific mAbs are expected to be acquired through the LassoGraft Technology^®^. Trispecific mAbs against TfR, EGFR, and, specifically, highly expressed cytosol molecules that are essential for cancer cells are the most potent targets to elicit anti-cancer activity in cancer cells. Microantibodies composed of the helix-loop-helix peptides (approximately 4 kDa), selected from a phage-displayed library [58] developed by Dr. Ikuo Fujii at Osaka Metropolitan University (Osaka, Japan), might be utilized for trispecific mAbs, in combination with LassoGraft Technology^®^.

However, such trispecific mAbs should escape from the endosomes or lysosomes to interact with the cytosol target molecules in brain cancer cells after the second round of endocytosis. This could be conducted via endosomal or lysosomal membrane rupture, based on the proton sponge effect, by introducing the polyamine unit. To briefly explain, weakly basic molecule-bound protons are absorbed by vacuolar H^+^-ATPase proton pumps. Protons continue to be absorbed as a result of this buffering effect. Osmotic swelling is induced by the entry of water molecules and chloride ions, accompanied by protons. Eventually, bursting occurred, releasing the endosomal or lysosomal contents into the cytoplasm. At the same time, such trispecific mAbs should not escape from endosomes and should separate from the TfR in endosomes in the capillary endothelial cells after the first endocytosis event. The affinity between anti-TfR trispecific mAbs with the polyamine unit and TfR should be relatively mild, to separate in the endosomes of the capillary endothelial cells under slightly weak acid conditions, even though the polyamine unit might accept a number of protons. Furthermore, the introduced polyamine unit should perform endosomal escape or lysosomal escape in brain cancer cells. Lysosomal membrane rupture might scatter the lysosomal enzymes all over the cytoplasm and may eventually induce cell death, even without carrying payloads. The escape potentiality of the polyamines could be tuned by varying the amine species. The pH in a late endosome is approximately 5.5 and the pH in a lysosome is approximately 4.5. Thus, when the pKa values of introduced polyamine units are adjusted to between 4.5 and 5.5, preferably between 4.5 and 5.0, lysosomes would be broken, based on the proton sponge effect. The pKa values of pyridine, aniline and 2-methylaniline at 25 °C in water are approximately 5.2, approximately 4.6, and approximately 4.7, respectively. Polyamines that are composed of pyridine derivatives or aniline derivatives are conceivable. Although pyridine derivatives or aniline derivatives are biologically toxic, they are buried under a large mAb protein and may not exhibit toxicity due to hindrance under normal conditions. In addition, units against the cytosol molecules essential to brain cancer cells are preferably relatively resistant to lysosomal enzymes, such as the cyclic peptides with D-amino acids or *N*-methyl amide groups, like enzymatically stable non-peptide cyclic Lys-MCC-DM1. Cyclosporin is an enzymatically stable cyclic peptide with D-amino acids and *N*-methyl amide groups and penetrates the membrane via passive diffusion, which suggests that special cyclic peptides with anti-cancer activity, obtained from the RaPID system, could passively cross the lysosomal membrane to the cytoplasm as a catabolite without needing transporters, such as SLC46A3. Nonetheless, a lysosome-specific cathepsin B-liable linker, such as valine-citrulline dipeptide, is cleaved in the lysosome [59]. Such enzymatically cleavable linkers should be used for ADCs with hydrophobic cancer drugs that can be transported across the lysosomal membrane via passive diffusion. As an intellectual challenge, inspired readers are expected to offer better suggestions.

## 3. Conclusions

The current brain cancer treatments are surgery, radiation therapy, and chemotherapy. It is difficult to remove all tumor cells, even with surgical removal after craniotomy. Thus, prognostic chemotherapy is very important. However, the BBB prevents drugs from entering the brain. Strategical drug delivery methods should be established to solve this impenetrable problem. RMT is one solution using receptors such as TfR or InsR that are expressed at the apical membrane of the capillary endothelial cells in the BBB. The delivery of cancer drugs into the brain via RMT can be developed using a system similar to J-Brain Cargo^®^ (Figure 5) [2,29], based on the conjugates of anti-TfR mAb and their cargoes. Moreover, drug delivery methods that can cross the BBB using anti-TfR mAb as a vector were reported widely, although they had not yet been used for brain cancers [Table 1]. Therefore, (i) ADCs using anti-TfR mAb as a vector and anti-cancer drugs as the payload cargo are promising agents for the treatment of brain cancers in a non-invasive way. Anti-TfR ADCs with linked low-molecular cargoes present an orthodox structural design (Figure 3). After TfR-mediated endocytosis, the pH of endosomes is gradually acidified by vacuolar H^+^-ATPase proton pumps, moving from a pH of approximately 6.5 in the early endosome to a pH of approximately 5.5 in the late endosome and, finally, in a pH of approximately 4.5 in the lysosome. In this acidified process, whereas the anti-TfR mAb-TfR complex was liberated, the pH-sensitive cleavable linkers were cleaved to liberate the anti-cancer drugs. Subsequent exocytosis released the free anti-cancer drugs to eventually fulfill their purpose. Alternatively, (ii) anti-TfR and anti-EGFR bispecific ADCs with low-molecular payloads present a well-defined design in terms of their strict distribution and limited side effects. As explained above, (i) anti-TfR ADCs with low-molecular anti-cancer drugs distributed via pH-sensitive cleavable linkers and (ii) anti-TfR and anti-EGFR bispecific ADCs with low-molecular anti-cancer drugs are implementable using the current technologies. Ultimately, (iii) trispecific mAbs that can work against TfR, EGFR, and cytosol molecules that are essential to brain cancer cells can be produced using LassoGraft Technology^®^. Intravenously administered, such trispecific mAbs with a polyamine unit would cross the endothelium at the BBB via RMT using TfR, and would then be endocytosed in target brain cancer cells through EGFR-mediated endocytosis and elicit anti-cancer activity after endosomal escape or lysosomal escape, probably due to membrane rupture from the proton sponge effect. However, endosomal escape should occur not in the capillary endothelial cells but in the brain cancer cells, acting by tuning the polyamine unit for optimization. Thus, well-coordinated drug designs are required in order to conduct the desired drug delivery and in order to show activity regarding the target molecules, by fully considering the related physically and biologically systematic structures. Multispecific mAbs created through LassoGraft Technology^®^ may play a vital role in biomedical therapy in the future, via pharmacokinetically precise distribution and pharmacodynamically high selectivity regarding target molecules.

It is not only macroscopic organic activity but also microscopic molecular behavior that is controlled by physically and biologically systematic structures, based on the structuralism proselytized by Lévi-Strauss, similar to one domino knocking over another or the way that the Monkey King, known as Sun Wukong, acted only in the palm of the Buddha. The activity and behavior of ions, DNA, RNA, proteins, organelles, cells, and organs as physical and biological substances under natural laws have largely been clarified. Substance interactions and trajectories can be predictable. The absorption, distribution, metabolism, elimination, and toxicity (ADMET) of a specific orally or intravenously administered drug can be conceived. Thus, drug delivery to target sites can be accomplished, based on a well-defined design, by following the universal rule in such structural pharmaceutical science, similar to the universal rule in structural linguistics developed by Saussure and that in the structural anthropology developed by Lévi-Strauss. Eventually, medicinal chemists and pharmaceutical scientists will produce innovative pharmaceutical agents, in order to cure brain cancers without the need for surgical removal via craniotomy.

## Figures and Tables

**Table 1:** All the compounds introduced in this perspective review.

#	Administrated Drug	Formulation/ Co-Administrated Drug	Disease	Vector	Cargo	Linker	Group	Status	References
(i)	Temozolomide	Low-molecular compound	Glioma	-	Temozolomide	-	-	Launched	[11]
(ii)	Bevacizumab	Anti-VEGFmAb	Glioma	-	Bevacizumab	-	-	Launched	[12]
(iii)	BCNU wafer	Low-molecular compound/polifeprosan 20 polymer	Glioma	-	BCNU	-	-	Launched	[13]
(iv)	Procarbazine	Low-molecular compound	Brain cancers	-	Procarbazine	-	-	Launched	[41]
(v)	Nimustine (ACNU)	Low-molecular compound	Brain cancers	-	Nimustine	-	Daiichi Sankyo	Launched	[42]
(vi)	Vincristine	Low-molecular compound	Brain cancers	-	Vincristine	-	-	Launched	[43]
(vii)	Tocilizumab (Actemra^®^)	Anti-IL-6 mAb	Rheumatoid arthritis	-	Tocilizumab	-	Chugai	Launched	[17]
(viii)	Nivolumab (Opdivo^®^)	Anti-PD-1 mAb	Metastatic lung squamous cell carcinoma	-	Nivolumab	-	Ono	Launched	[18]
(ix)	Mogamulizumab (Poteligeo^®^)	Anti-CCR4 mAb	Relapsed or refractory mycosis fungoides and Sézary disease	-	Mogamulizumab	-	Kyowa Kirin	Launched	[19]
(x)	Burosumab (Crysvita^®^)	Anti-FGF23 mAb	X-linked hypophosphatemic rickets	-	Burosumab	-	Kyowa Kirin	Launched	[20]
(xi)	Trastuzumab deruxtecan (Enhertu^®^)	Anti-HER2 ADC	HER2 positive breast cancer	Anti-HER2 mAb	Deruxtecan	Linker	Daiichi Sankyo	Launched	[21,23]
(xii)	Datopotamab deruxtecan (Dato-DXd)	Anti-ROP2 ADC	Solid cancers	Anti-ROP2 mAb	Deruxtecan	Linker	Daiichi Sankyo	Clinical trial	[24]
(xiii)	Patritumab deruxtecan (HER3-DXd)	Anti-HER3 ADC	Solid cancers	Anti-HER3 mAb	Deruxtecan	Linker	Daiichi Sankyo	Clinical trial	[25]
(xiv)	DS-7300	Anti-B7-H3 ADC	Solid cancers	Anti-B7-H mAb	Deruxtecan	Linker	Daiichi Sankyo	Clinical trial	[26]
(xv)	DS-6000	Anti-CDH6 ADC	Solid cancers	Anti-CDH6	Deruxtecan	Linker	Daiichi Sankyo	Clinical trial	[27]
(xvi)	DS-3939	Anti-TA-MUC1 ADC	Solid cancers	Anti-TA-MUC1	Deruxtecan	Linker	Daiichi Sankyo	Pre-clinical	[27]
(xvii)	Idursulfase beta	Anti-TfR ADC with-iduronate-2-sulfatase	Hunter syndrome	Anti-TfR mAb	Iduronate-2-sulfatase	Fusion protein	JCR Pharmaceuticals	Launched	[30]
(xviii)	Bispecific RmAb158-scFv8D3	Bispecific RmAb158-scFv8D3	Alzheimer’s disease	Anti-TfR scFv8D3	Anti-soluble AβRmAb158	Linker	-	Basic research	[31]
(xix)	Anti-TfR mAb OX26 variant with galanin	Anti-TfR ADC with galanin	Induction of homeostatic rebound sleep	Anti-TfR mAb	Neuropeptide galanin	Linker	-	Basic research	[32]
(xx)	Anti-TfR and anti-BACE1 bispecific mAb	Anti-TfR and anti-BACE1 bispecific mAb	Alzheimer’s disease	Anti-TfR mAb	Anti-BACE1 mAb	Fusion protein	-	Basic research	[33]
(xxi)	Anti-TfR mAb with erythropoietin	Anti-TfR mAb fused to erythropoietin	Alzheimer’s disease	Anti-TfR mAb	erythropoietin	Fusion protein	-	Basic research	[34]
(xxii)	scFab of anti-TfR mAb and anti-Aβ mAb31	scFab of anti-TfR mAb and anti-Aβ mAb31	Alzheimer’s disease	scFab of anti-TfR mAb	anti-Aβ mAb31	Linker	-	Basic research	[35]
(xxiii)	ABT-414 (depatuxizumab mafodotin)	Anti-EGFR ADC with MMAF	Glioblastoma	Depatuxizumab	MMAF	Linker	-	Basic research	[46]
(xxiv)	AMG-595	Anti-EGFR ADC with DM1	Glioblastoma	Anti-EGFR mAb	DM1 (mertansine)	Linker	-	Basic research	[47]
(xxv)	Aducanumab	Anti-Aβ mAb	Alzheimer’s disease	-	Anti-Aβ mAb	-	Eisai	Launched	[9,49]
(xxvi)	Emicizumab (Hemlibra^®^)	Anti-blood coagulation factor IX and X bispecific mAb	Hemophilia A	-	Anti-blood coagulation factor IX and X bispecific mAb		Chugai	Launched	[57]
(xxvii)	Anti-TfR ADCs with linked low-molecular cargos	Anti-TfR ADCs with linked low-molecular cargos	Glioma	Anti-TfR mAb	Brain cancer drugs	pH-sensitive cleavable linker	Tashima lab	Under analysis in Tashima lab	-
(xxviii)	Anti-TfR and anti-EGFR bispecific ADCs with low-molecular payloads	Anti-TfR and anti-EGFR bispecific ADCs with low-molecular payloads	Glioma	Anti-TfR mAb Anti-EGFR mAb	Brain cancer drugs	Linker	Tashima lab	Under analysis in Tashima lab	-
(xxix)	Trispecific mAbs against TfR, EGFR, and tumor-specific molecules	Trispecific mAbs against TfR, EGFR, and tumor-specific molecules	Glioma	Anti-TfR and anti-EGFR mAbs	Anti-tumor-specific molecules unit	-	Tashima lab	Under analysis in Tashima lab	-

## Data Availability

Not applicable.
